# The Role of Heart Team Approach in Penetrating Cardiac Trauma: Case Report and Review of the Literature

**DOI:** 10.21470/1678-9741-2017-0150

**Published:** 2018

**Authors:** Marzia Cottini, Amedeo Pergolini, Federico Ranocchi, Francesco Musumeci

**Affiliations:** 1 Department of Heart and Vessels, Cardiac Surgery Unit and Heart Transplantation Center, "S. Camillo-Forlanini" Hospital, Rome, Italy.

**Keywords:** Wounds, penetrating, Heart septal defects, ventricular, Cardiac Tamponade, Cardiac surgical procedures, Heart injuries, Septal occluder device

## Abstract

Penetrating cardiac trauma has been increasing in clinical experience and is joined to important morbidity and mortality. A case of a 38-year-old female with history of postpartum depression was reported, admitted to our department for cardiac tamponade due to penetrating self-inflicted multiple stab wound of the chest complicated by rupture of anterior left ventricular wall and traumatic ventricular septal defect. Following the unstable hemodynamic instability, a combined therapeutic strategy was chosen: surgery and transcatheter implantation to correct free wall ventricle damage and traumatic ventricular septal defect, respectively.

**Table:** 

Abbreviations, acronyms & symbols	
CVP	= Central venous pressure
ECG	= Echocardiography
TTE	= Transthoracic echocardiogram
tVSD	= Traumatic ventricular septal defect
VSD	= Ventricular septal defect

## INTRODUCTION

A very rare and uncommon case of penetrating cardiac injuries was reported, due to multiple self-inflicted stabs in a young female with a history of postpartum depression and causing cardiac tamponade due to free ventricular wall rupture and iatrogenic ventricular septal defect. The particularity of combined therapeutic choices in the same time - surgical drainage of blood pericardial effusion and endovascular closure of traumatic ventricular septal defect (tVSD) with ventricular septal defect (VSD) occluder device - was described.

## CASE REPORT 

**Fig. 1:**
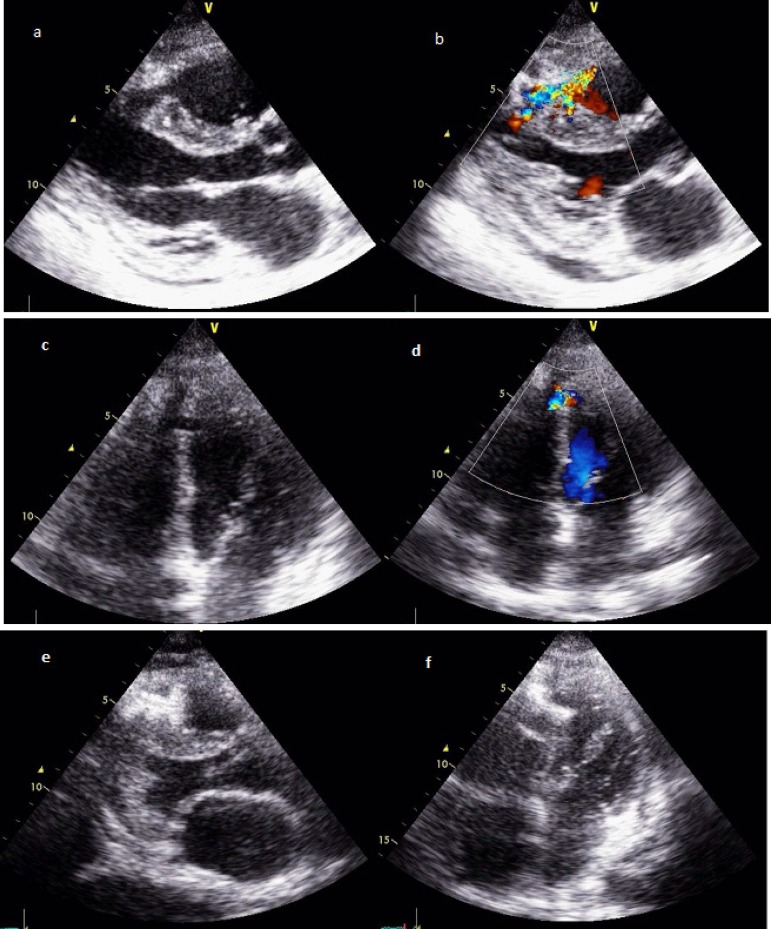
Transthoracic echocardiography, parasternal view showing interventricular traumatic defect in the median septum (a), and evidence of left-to-right shunt (b). Transthoracic echocardiography, apical view of iatrogenic interventricular defect (c) and color-doppler image of the L-R shunt (d). Transthoracic echocardiography, longitudinal view of the successful implanting Amplatzer device to close the tVSD (e-f ).

**Fig. 2:**
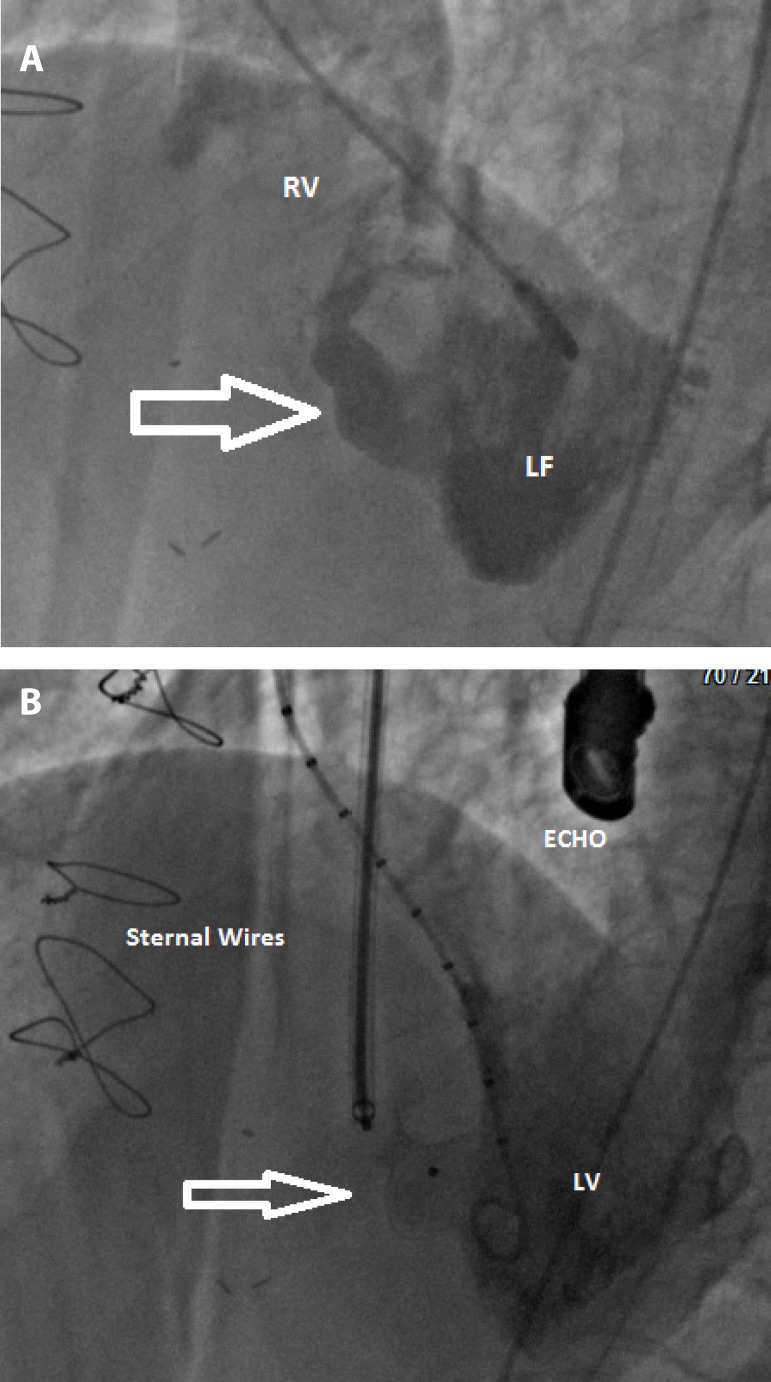
Conventional coronary angiography documented traumatic ventricular septal defect (a) and positioning of VSD occluder (white arrow) with good results (b).

A case of a 38-year-old female with a history of post-partum depression was presented, referring to our hospital for penetrating self-inflicted multiple stab wounds of the chest. Vital signs of arrival were systolic blood pressure of 80/45 mmHg, tachypnea (30 breaths/min) with low oxygen saturation (89%), cyanosis and jugular vein distension (central venous pressure of 15-16 cmH2O). The echocardiography (ECG) documented raised ST, J waves. The fast-transthoracic echocardiogram (TTE) revealed a cardiac tamponade (maximum diameter 3.2 cm) and a VSD (about 1.5-1.8 cm from left ventricle side) with ventricular left-right shunt (Qp:Qs=2, Figures 1A to D). Following the critical hemodynamic deterioration, the norepinephrine and epinephrine infusions were started (0.1 mcg/kg/min) and the patient was immediately operated. Combined unusual therapeutic strategy has been chosen: surgery for the pericardial effusion drainage and control of the ventricular wall wounds, and transcatheter closure of the tVSD. Median sternotomy and a T-inverted pericardiotomy were performed to remove all clots and pericardial effusion from mediastinum. After the detection of the heart, we found a single left ventricle anterior wall wound, hence we directly closed with a direct suture (3-0 prolene with Teflon pledgets). To complete the treatment, the patient underwent a procedure to position the Amplatzer VSD occluder device (16-mm) by transcatheter way in the same operation time (Figures 1E to F and Figure 2B). The procedures were free of complications and the postoperative period was short and uneventfully. She was discharged on the 10^th^ postoperative day with single antiaggregation therapy and was followed-up by psychological support service.

## DISCUSSION 

The major cardiac injuries could be blunt or penetrating. Penetrating cardiac trauma has different and several presentations^[1-10]^. The patient could be presented with a stable tamponade (hypotension, elevated central venous pressure [CVP]) or unstable ones (shock with critical hypotension, tachycardia, dyspnoea, raised CVP, pulsus paradoxus with distant heart sounds and impalpable apex). Our patient fitted in the unstable patient type: the decision needed to be made very quickly^[7-15]^. The first step was the diagnostic workout (chest X-ray, ECG, computed tomography scan, TTE) that identify and describe the size, type and setting of the lesion and general assessment. In our case, the patient had a cardiac tamponade due to stab complicated to iatrogenic VSD. Therefore, we decided to proceed with combined therapeutic path in the same time: 1) surgical approach to suture ventricular wound and 2) endovascular approach to close iatrogenic VSD with an occluder device. This case is the first reported in scientific literature because the most of the previous article describe single procedure for closure of VSD with occluder device after surgery or only endovascular approach or first endovascular and then surgery correction, but there are not combined procedures in the same time (Table 1), following some of the most important experience in the literature. According to Degiannis et al.^[21]^, surgical approach could be fundamental and the primary step to control the bleeding, in particularly the best is median sternotomy approach which gives an effective and extensive vision of heart, great vessels, to other structures in the mediastinum and to both pleural cavities^[1-9,13-16]^. On the other hand, left antero-lateral thoracotomy provides rapid access to the right and left ventricles and to the pulmonary artery; this is our approach of choice for emergency room thoracotomy^[21]^. In case of penetrating cardiac injuries complicated by iatrogenic VSD, the combined therapeutic choice with surgery and percutaneous device was described by Argento et al.^[12]^, in 2002. Afterwards, only three cases by Berry et al.^[13]^ and Ali et al.^[17]^, with good results (low postoperative recovery, total cardiac function restore without any interventricular septum shunt) were published. The use of minimally surgery (opening, controlling and treating the cause of bleeding) associated with the percutaneous occluder device implantation in penetrating cardiac injuries with iatrogenic VSD may be a complete and safe approach to this trauma patient. The tVSD exclusion by percutaneous device avoided long surgical timing hence less invasiveness, no cardiopulmonary bypass, less anesthesia time and recovery time.

**Table 1:** Review of previous ventricular septal defect (VSD) after cardiac wound stab described in scientific literature and their treatments. The traumatic VSD was diagnosed immediately, deferred VSD diagnosis was not considered.

Author	Year	Type of Paper	Patient	Gender	Complication	Therapeutic choice
Lui et al.^[1]^	1965	CR	1	Male	Cardiac tamponade	Surgery
Pejaković & Mileusnić^[2]^	1967	CR	1	Male	Cardiac tamponade	Surgery
Kieny et al.^[3]^	1975	CR	1	Male	Cardiac tamponade	Surgery
Asfaw et al.^[4]^	1975	RL	12	Male	HF, injury of tricuspid valve, injury of left anterior descending coronary artery	Surgery
Bande et al.^[5]^	1980	CR and RL	1	Male	Cardiac tamponade	Surgery
Bryan et al.^[6]^	1988	CR	1	Male	Cardiac tamponade	Surgery
Voronov et al.^[7]^	1989	CR	1	Male	Cardiac tamponade	Surgery, suture
Take et al.^[8]^	1993	CR	1	Female	Rupture of papillary muscle	Surgery
Carvalho et al.^[9]^	1994	CR	1	Male	Hemothorax	Surgery, patch suture
Doty et al.^[10]^	1999	CR	1	Male	Tricuspid valve injury	Surgery
Gölbasi et al.^[11]^	2001	CR	1	Male	Cardiac tamponade	Surgery, suture
Argento et al.^[12]^	2002	CR	1	Male	Cardiac tamponade	Thoracotomy and percutaneous device
Berry et al.^[13]^	2006	CR	1	Male	Cardiac tamponade	Surgery and percutaneous device
Topaloglu et al.^[14]^	2006	CR	1	Male	Cardiac tamponade	Surgery
Choi et al.^[15]^	2008	CR and RL	1	Male	Atrioventricular valves rupture	Surgery
Antoniades et al.^[16]^	2011	CR	1	Female	Pneumothorax and cardiac tamponade	Surgery
Ali et al.^[17]^	2013	CR	1	Male	Cardiac tamponade	Surgery and percutaneous device
Caffery et al.^[18]^	2014	CR	1	Male	Hemothorax	Percutaneous device then surgery
Tang et al.^[19]^	2016	CR	1	Male	Congestive heart failure	Percutaneous device then surgery
Kharwar et al.^[20]^	2016	CR	1	Male	Cardiac tamponade	Percutaneous device
Cottini et al. (reported case)	2018	CR and RL	1	Female	Cardiac tamponade	Surgery and percutaneous device

CR=case report; RL=review of the literature; HF=heart failure

## CONCLUSION 

The combined therapeutic choice of surgery and interventional approach in case of penetrating cardiac trauma with limited tVSD is indicated and optimal for rapid clinical stabilization.

The rapid and early diagnosis associated with an organized and available cardiac staff (interventional cardiologists and cardiac surgeons) may be a productive collaboration.

**Table:** 

Authors' roles & responsibilities	
MC	First Author, revision and corresponding author; final approval of the version to be published
AP	Writting and revision; final approval of the version to be published
FR	Revising; final approval of the version to be published
FM	Revised critically; final approval of the version to be published
